# Supplementary material for: Differential Responses of Bacterial and Fungal Communities to Siderophore Supplementation in Soil Affected by Tobacco Bacterial Wilt (Ralstonia solanacearum)
Source: Microorganisms. 2023 Jun 9;11(6):1535. doi: 10.3390/microorganisms11061535 (PMC10302624; doi:10.3390/microorganisms11061535)
Supplement: Supplementary file 1 [file microorganisms-11-01535-s001.zip › Figure S1.pdf]

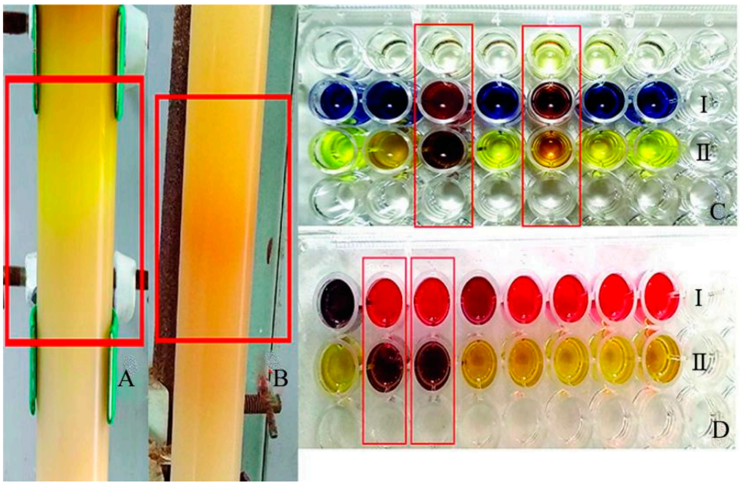


**Figure S1** Separation of siderophore active fractions from 2-14F2 and 2-8F2 strains. A-B:Sephadex-LH20 gel segmentation for 2-14F2 and 2-8F2 strains;c-d: CAS and $FeCl_3$ detection using 96-well plate for Sephadex-LH20 gel segmentation of 2-14F2 and 2-8F2 strains (I is the test result by CAS, II is the test result by 2% $FeCl_3$)
