# Supplementary material for: Differential Responses of Bacterial and Fungal Communities to Siderophore Supplementation in Soil Affected by Tobacco Bacterial Wilt (Ralstonia solanacearum)
Source: Microorganisms. 2023 Jun 9;11(6):1535. doi: 10.3390/microorganisms11061535 (PMC10302624; doi:10.3390/microorganisms11061535)
Supplement: Supplementary file 1 [file microorganisms-11-01535-s001.zip › Figure S3.pdf]

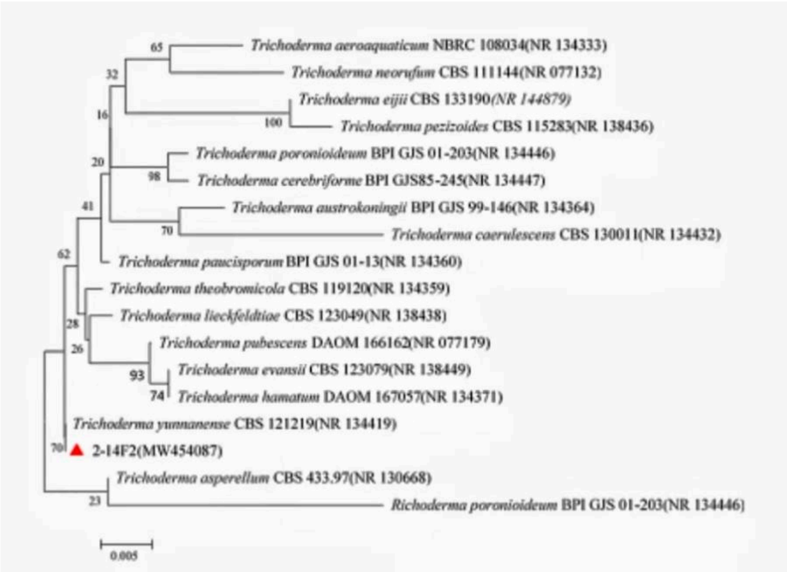

Figure S3 NJ tree based on ITS sequences showing the phylogenetic relationships between strain 2-14F2 and representative members of *Trichoderma*. Bootstrap values (> 50%) based on 1000 replicates were shown at the branch nodes. *Richoderma poronioideum* BPI GJS 01-203 is used as an outgroup. Bar, 0.5% sequence divergence.
