# Supplementary material for: Differential Responses of Bacterial and Fungal Communities to Siderophore Supplementation in Soil Affected by Tobacco Bacterial Wilt (Ralstonia solanacearum)
Source: Microorganisms. 2023 Jun 9;11(6):1535. doi: 10.3390/microorganisms11061535 (PMC10302624; doi:10.3390/microorganisms11061535)
Supplement: Supplementary file 1 [file microorganisms-11-01535-s001.zip › Table S1.pdf]

Table S1. Siderophore type and siderophore units of different strains.

Species	Siderophore Type	Siderophore Units (%)	References
Beauveria sp. (2-8F2)	Hydroxamaces	62.02	This study
Beauveria brongniartii	Hydroxamaces	59.81	Ref. [30]
Trichoderma sp. (2-14F2)	Hydroxamaces	52.06	This study
Trichoderma asperellum	Hydroxamaces	38.62	Ref. [32]
Trichoderma harzianum	Hydroxamaces	50.63	Ref. [33]
Trichoderma virens	-	40.23	Ref. [34]
Trichoderma peudokoningii	Hydroxamaces	50.43	Ref. [35]
